# Introduction of a sexual health practice nurse is associated with increased STI testing of men who have sex with men in primary care

**DOI:** 10.1186/1471-2334-13-298

**Published:** 2013-07-01

**Authors:** Anthony F Snow, Lenka A Vodstrcil, Christopher K Fairley, Carol El-Hayek, Rosey Cummings, Louise Owen, Norman Roth, Margaret E Hellard, Marcus Y Chen

**Affiliations:** 1 Melbourne Sexual Health Centre, Alfred Health, Carlton, Victoria, Australia; 2 School of Population Health, University of Melbourne, Parkville, Victoria, Australia; 3 Centre for Population Health, Burnet Institute, Melbourne, Victoria, Australia; 4 Centre Clinic, Victorian AIDS Council/Gay Men’s Health Centre, St Kilda, Victoria, Australia; 5 Prahran Market Clinic, Prahran, Victoria, Australia

**Keywords:** Primary health care, Nursing, Men who have sex with men, HIV, Sexually transmitted infections, Screening

## Abstract

**Background:**

The study objective was to investigate the effect of the introduction of a sexual health practice nurse on HIV and STI testing in a general practice that specialized in gay men’s health.

**Methods:**

This observational study compared the proportion of gay and other men who have sex with men (MSM) tested for HIV, syphilis, chlamydia (urethral and anal) and gonorrhoea (anal), or all of the above (defined as a complete set of tests at a single visit), two years before and one year after the nurse was introduced (Clinic A). Clinic B, a general practice which also specialized in gay men’s health, but with no sexual health nurse, was used as a control.

**Results:**

In Clinic A, amongst HIV negative MSM the proportion of men who had a complete set of HIV and STI tests increased from 41% to 47% (p < 0.01) after the nurse was introduced. Amongst HIV positive MSM attending clinic A there was an increase in the proportion of men who had a complete set of tests after the nurse was introduced from 27% to 43% (p < 0.001). In Clinic B there was no significant increase in testing in the proportion of either HIV negative or HIV positive men who had a complete set of tests over the same time periods.

**Conclusions:**

The introduction of the sexual health practice nurse resulted in significant increases in episodes of complete STI testing among MSM. The effect was most pronounced among HIV positive MSM.

## Background

Gay and other men who have sex with men (MSM) constitute a major risk group for Human Immunodeficiency Virus (HIV) and sexually transmitted infections (STIs) [1]. Increased HIV and STI testing could enhance the control of these often, asymptomatic infections among MSM [2-5]. Australian guidelines recommend MSM should have at least annual screening for urethral and rectal chlamydia, pharyngeal and rectal gonorrhea, syphilis and HIV with more frequent testing for those at higher risk [6]. Self-reported data from gay community periodic surveys indicates the majority of HIV negative and HIV positive men attended their regular general practitioner (GP) for their last syphilis test [7]. Furthermore, a survey of people living with HIV has found almost half of respondents attended a GP for HIV management and treatment [8]. However, there is evidence that testing rates in this population is sub-optimal [9]. Data from three Melbourne general practices with high caseloads of MSM indicated sub-optimal testing rates among MSM, particularly among those at higher risk [10]. Furthermore, MSM in Melbourne and Sydney self-report lower rates of complete HIV and STI testing compared with being tested for any STI test [11].

There are limited published studies on clinic-based interventions that have been shown to increase HIV and STI testing among MSM [12]. While nurse led STI screening within sexual health clinic setting is common place [13-15], to our knowledge, there have been no published studies that have examined the efficacy of sexual health nurses in the general practice setting to improve HIV and STI testing among MSM in particular.

In October 2008, an experienced sexual health practice nurse was introduced into an urban Melbourne general practice specializing in gay men’s health. The aim of this intervention was to enhance HIV and STI testing at the clinic, which provided general practice, sexual health and HIV treatment to its mainly MSM patient population. Prior to the introduction of the sexual health nurse the clinic did not employ a practice nurse. The overall objective of the current study was to determine the effect the introduction of the sexual health practice nurse (i.e. the intervention) had on HIV and STI testing. We evaluated the number of HIV and STI tests as a proportion of MSM who attended the clinic before and after the intervention.

## Methods

### Setting

We undertook a study comparing the proportion of men who were tested for HIV and STIs before and after a sexual health practice nurse was introduced into a clinic (Clinic A) with the aim of increasing HIV and STI testing. A comparison clinic (Clinic B), a general practice also specializing in gay men’s health, but with no sexual health nurse, was used as a control clinic. At Clinic A, clients were able to access the sexual health nurse for testing either by appointment, or directly following a consultation with their general practitioner if they recommended testing. Clinic A operated utilizing the Australian government universal insurance scheme, Medicare, with no direct costs to clients. Although Clinic B also used Medicare, clients had to pay some additional consultation fees. In addition, the sexual health nurse provided HIV chronic disease care, whereby HIV positive MSM could visit the nurse every three months for a range of services, including, but not limited to, the collection of routine HIV serologic monitoring tests and STI testing. At Clinic B, all HIV and STI testing was carried out by general practitioners only. At each clinic the routine practice was for patients to self-collect anal and urine samples.

### Study design

We compared the proportion of men who were tested at each clinic in the 24 months prior to the intervention (Period 1: 1^st^ October 2006 to 30^th^ September 2007; and Period 2: 1^st^ October 2007 to 30^th^ September 2008) and the 12 months after (Period 3: 1^st^ October 2008 to 30^th^ September 2009) the sexual health nurse was introduced. The two periods prior to the intervention, Periods 1 and 2 were included to establish whether there was a background increase in testing prior to the introduction of the nurse.

### Data extraction

The total number of MSM attending each clinic was retrieved from each clinics electronic patient database. The number of HIV positive MSM attending each clinic was obtained by counting the number of men who had at least one HIV viral load assay in each period. Men who had an HIV viral load test performed at any visit over the three periods were included as HIV positive. However, if an individual had a negative HIV antibody test at an earlier visit before they had HIV viral load tests, they were deemed to be HIV negative for any prior visits.

HIV viral load tests and the number of HIV and STI tests undertaken during these periods and at both clinics was obtained from the Victorian Primary Care Network for Sentinel Surveillance of STIs and Blood Borne Viruses [16]. The tests included serology for HIV antibodies and syphilis enzyme-linked immunoassay (EIA), a urine specimen for chlamydia; and a rectal swab for chlamydia and gonorrhoea. The Victorian Primary Care Network for Sentinel Surveillance of STIs and Blood Borne Viruses [16] does not routinely collect data for pharyngeal gonorrhoea testing; therefore it was not included in the analysis. An episode of complete testing was defined as the collection of serology for HIV antibodies (for HIV negative MSM); syphilis EIA; a urine test; and an anal swab on the same date.

### Analysis

MSM were stratified by HIV status and analyzed separately using SPSS v.18. The proportion of men who were tested for each STI or for an episode of complete testing was calculated for each clinic and time periods. If an individual attended and was tested more than once in the same period he was only counted once. Chi-squared (χ^2^) was used to test for differences in proportions of men who had each test or a complete episode of testing. If a man presented in multiple periods the visits were considered to be independent observations and so were included once for every period they attended in. The Alfred Hospital Research Ethics Committee granted approval for this study.

## Results

### HIV and STI testing in HIV negative men

Among HIV negative MSM at Clinic A (Table 1), there was no significant difference in the proportion of men tested for HIV, syphilis, or who had a urine test, an anal swab or a complete episode of testing from Periods 1 to Period 2. When Compared to Period 2, the introduction of the sexual health nurse in Period 3 (Table 1) was associated with significant increases in the proportion of HIV negative MSM who had HIV, syphilis, urine, anal, or complete sets of tests with absolute increases of 5% (p = 0.026), 7% (p < 0.01), 5% *( p =* 0.024), 7% (p < 0.01), and 6% (p < 0.01) respectively.

**Table 1:** Comparison of HIV and STI testing of HIV negative MSM attending two general practices during three one-year periods

**Clinic A: Nurse introduced in Period 3**	**n**	**n**_**t**_**(%)**	**95% CI**	**% Difference Period 1 to Period 2**	**% Difference Period 2 to Period 3**
**HIV antibody**					
Period 1	1000	504 (50)	47-54		
Period 2	1011	523 (52)	49-55	*2 ( p* = 0.39)	
Period 3	1042	596 (57)	54-60		*5 ( p* = 0.026)
**Syphilis EIA**^**#**^					
Period 1	1000	522 (52)	49-55		
Period 2	1011	534 (53)	50-56	*1( p* = 0.69)	
Period 3	1042	630 (60)	57-63		*7 ( p* < 0.01)
**Urine Test**					
Period 1	1000	526 (53)	50-56		
Period 2	1011	572 (57)	54-60	*4 (p* = 0.08)	
Period 3	1042	649 (62)	59-65		*5 ( p =* 0.024)
**Anal Swab**					
Period 1	1000	477 (48)	45-51		
Period 2	1011	506 (50)	47-53	*2 (p* = 0.39)	
Period 3	1042	590 (57)	54-60		*7 (p* < 0.01)
Complete Tests*					
Period 1	1000	383 (38)	35-41		
Period 2	1011	411 (41)	38-44	*3 (p* = 0.18)	
Period 3	1042	489 (47)	44-50		*6 ( p* < 0.01)
**Clinic B: No Nurse**	**n**	**n**_**t**_**(%)**	**95% CI**	**% Difference Period 1 to Period 2**	**% Difference Period 2 to Period 3**
**HIV antibody**					
Period 1	3664	1429 (39)	37- 41		
Period 2	3836	1643 (43)	41- 45	*4 ( p* < 0.001)	
Period 3	3870	1442 (37)	35- 39		*−6 (p* < 0.001)
**Syphilis EIA**^**#**^					
Period 1	3664	1498 (41)	39- 43		
Period 2	3836	1711 (45)	43- 47	*4 (p* < 0.001)	
Period 3	3870	1549 (40)	38- 42		*−5 (p* < 0.001)
**Urine Test**					
Period 1	3664	1345 (37)	35-39		
Period 2	3836	1542 (40)	38-42	*3 (p* < 0.001)	
Period 3	3870	1369 (35)	34-36		*−5 ( p* < 0.01)
**Anal Swab**					
Period 1	3664	922 (25)	22-26		
Period 2	3836	1148 (30)	29- 31	*5 ( p* < 0.001)	
Period 3	3870	1022 (26)	25- 27		*−4 (p* < 0.001)
Complete Tests*					
Period 1	3664	755 (21)	20-22		
Period 2	3836	971 (25)	24-26	*4 (p* < 0.001)	
Period 3	3870	883 (23)	22-24		*- 2 (p* < 0.01)

n = number of MSM.

n_t =_ number of test.

^**#**^ EIA = Enzyme-linked Immunoassay.

*Complete Tests = HIV antibodies; syphilis EIA; urine test for chlamydia and an anal swab for chlamydia and gonorrhoea collected on the same date.

Among HIV negative MSM who attended Clinic B (Table 1), between Period 1 and Period 2 there were significant increases, ranging from 3% to 5% (all p < 0.001), in the proportion of men tested for all tests including episodes of complete testing. However, between Period 2 and Period 3 there were significant decreases in the proportion of HIV negative men tested at Clinic B for all tests including episodes of complete testing. From Period 2 to Period 3 the decrease in the proportion of men tested ranged from 2% for episodes of complete testing to 6% for HIV testing (all p < 0.001).

### STI Testing in HIV positive men

For HIV positive MSM who attended Clinic A (Table 2), there was no significant change in the proportion tested for syphilis from Period 1 to Period 2 (p = 0.09), or from Period 2 to Period 3 (p = 0.2). For this group there were no significant differences in the proportion of men who had a urine test (p = 0.12), an anal swab (p = 0.30), or complete testing (p = 0.83) from Period 1 to Period 2. Compared with Period 2, the introduction of the sexual health practice nurse in Period 3 (Table 2) was associated with a significant increase in the proportion of HIV positive men who had a urine test, an anal swab or complete testing: by 8% (p = 0.029), 11% (p < 0.01) and 16% (p < 0.001) respectively. The significant increase in the proportion of anal swabs performed is likely to be the main cause of the subsequent increase in complete episodes of testing.

**Table 2:** Comparison of STI testing of HIV positive MSM attending two general practices during three one-year periods

**Clinic A: Nurse introduced in Period 3**	**n**	**n**_**t**_**(%)**	**95% CI**	**% Difference Period 1 to Period 2**	**% Difference Period 2 to Period 3**
**Syphilis EIA**^**#**^					
Period 1	346	296 (86)	82-89		
Period 2	374	303 (81)	77-85	*−4 (p* = 0.09)	
Period 3	418	321 (77)	73-81		*−4 (p* = 0.20)
**Urine Test**					
Period 1	346	135 (39)	34-44		
Period 2	374	169 (45)	40-50	*6 (p* = 0.12)	
Period 3	418	222 (53)	46-60		*8 (p* = 0.029)
**Anal Swab**					
Period 1	346	120 (35)	30-40		
Period 2	374	146 (39)	34-44	*4 (p* = 0.30)	
Period 3	418	208 (50)	45-55		*11 (p* < 0.01)
**Urine and Anal Tests**					
Period 1	346	109 (32)	27-37		
Period 2	374	138 (37)	32-42	*5 (p* = 0.18)	
Period 3	418	201 (48)	43-53		*11 (p* < 0.01)
Complete Tests*					
Period 1	346	89 (26)	22-31		
Period 2	374	100 (27)	23-32	*1 (p* = 0.83)	
Period 3	418	180 (43)	38-48		*16 (p* < 0.001)
**Clinic B: No Nurse**	**n**	**n**_**t**_**(%)**	**95% CI**	**% Difference Period 1 to Period 2**	**% Difference Period 2 to Period 3**
**Syphilis EIA**^**#**^					
Period 1	773	652 (84)	81-86		
Period 2	830	723 (87)	84-89	*3 (p* = 0.13)	
Period 3	858	711 (83)	80-85		*−4 (p* = 0.02)
**Urine Test**					
Period 1	773	140 (18)	15-21		
Period 2	830	182 (22)	19-25	*4 (p* = 0.17)	
Period 3	858	185 (22)	19-25		*0 (p* = 0.31)
**Anal Swab**					
Period 1	773	106 (16)	13-19		
Period 2	830	133 (18)	15-21	*2 (p* = 0.07)	
Period 3	858	137 (19)	16-22		*1 (p* = 0.90)
**Urine and Anal Tests**					
Period 1	773	79 (10)	8-12		
Period 2	830	120 (14)	12-17	*4 (p* = 0.013)	
Period 3	858	126 (15)	13-18		*1 (p* = 0.95)
**Complete Tests**					
Period 1	773	46 (6)	4-8		
Period 2	830	65 (8)	6-10	*2 (p* = 0.17)	
Period 3	858	80 (9)	7-11		*1 (p* = 0.31)

n = number of MSM.

n_t =_ number of tests.

^**#**^EIA = Enzyme-linked Immunoassay.

*Complete Tests = HIV antibodies; syphilis EIA; urine test for chlamydia and an anal swab for chlamydia and gonorrhoea collected on the same date.

At Clinic B, there was no significant difference in the proportion of HIV positive MSM tested for syphilis (p = 0.13) from Period 1 to Period 2, and a small but statistically significant decrease (p = 0.02) from Period 2 to Period 3 (Table 2). The were no significant differences in the proportion of men who had a urine test, an anal swab or complete testing from Period 1 to Period 2, or from Period 2 to Period 3.

## Discussion

To our knowledge, this is the first published study examining the effect of the introduction of a sexual health practice nurse into general practice setting on HIV and STI testing among MSM. Introduction of the nurse was associated with significant increases in HIV and STI testing among HIV negative MSM, and STI testing among HIV positive MSM. The only exception to this was syphilis testing among HIV positive MSM, which did not significantly change. In the control clinic, where no sexual health nurse was introduced, we found a significant decrease in the proportion of HIV negative MSM tested for HIV and STIs over the same period. With the exception of syphilis, we found no significant change in STI testing among HIV positive MSM.

Importantly, there was an increase in the proportion of HIV negative and HIV positive MSM who had complete testing following the introduction of the nurse into the intervention clinic. This effect was most pronounced in HIV positive MSM, where episodes of complete testing increased by 16% (p < 0.001) – from 27% to 43%; whereas episodes of complete testing in the control clinic did not significantly change. Furthermore, at the intervention clinic, after the nurse was introduced, the proportion of HIV negative men tested for HIV increased by 5% - from 52% to 57% (p = 0.026). At the control clinic, the proportion of HIV negative men tested for HIV decreased by 6% - from 43% to 37% (p < 0.001).

Among HIV positive MSM in the intervention clinic, there was no significant change in syphilis testing (p = 0.20) after the sexual health nurse was introduced. There was a small but statistically significant (p = 0.02) decrease in syphilis testing among HIV positive MSM at the control clinic over the same period. However, this decrease may not be of clinical significance, given the high portion of HIV positive men tested for syphilis across all three periods in both clinics. The most likely reason for the high proportion of HIV positive men tested for syphilis is that prior to the commencement of this study, and throughout the study periods, both clinics had a policy in place to testing HIV positive MSM for syphilis with each routine blood test taken as part of HIV monitoring. This policy was in line with the recommendations of Australian National Gay Men’s Syphilis Action Plan [17].

The findings of our study indicate the introduction of the sexual health nurse enhanced HIV and STI testing not only in relation to the proportion of men tested, but also in terms of increased episodes of complete testing as recommended by testing guidelines [6]. This is important because increased STI screening of MSM has been shown to increase detection of infection [18]. There are number of possible reasons for these findings. First, GPs may have been more likely to initiate testing, knowing the nurse was available to carry out the tests, without testing impacting on their own consultation time [19]. Second, the nurse may have had more time with clients and was more likely to adhere to testing guidelines [19]. Third, the introduction of the nurse enabled increased access to service [19].

A limitation of this study is that changes in testing could have been affected by factors other than the introduction of the sexual health nurse. The Australian MSM STI screening guidelines were first published in 2002, approximately six years prior to the introduction of the nurse into the intervention clinic, and have since undergone biennial review [20]. Given the clinical focus of the clinics’ in our study, coupled with their respective policies, based on the Australian National Gay Men’s Syphilis Action Plan [17], it is not unrealistic to infer that GPs had a high awareness of the STI testing guidelines prior to the introduction of the nurse into the intervention clinic. Therefore, it is unlikely that there was a sudden or increasing awareness of the STI testing guidelines operating among the GPs at the intervention clinic which could account for the increases in testing which we found after the nurse was introduced. Furthermore, a social marketing campaign ‘Drama Downunder’, which ran throughout 2008 and 2009 aimed to promote HIV and STI testing in Victorian MSM [21]. However, this is unlikely to account for the significant increases in episodes of complete testing which were observed following the introduction of the sexual health practice nurse at the intervention clinic.

Ideally, future research would incorporate behavioral data, to stratify individual sexual risk behavior and frequency of testing. Future research should also include the effect on detection of infection. Furthermore, a cost-benefit evaluation needs to be undertaken, to assess the sustainability of this intervention.

## Conclusions

The increases in testing associated with the introduction of the nurse were achieved above already comparatively high baseline rates of testing. The effect of this intervention could therefore be even more pronounced in general practices where HIV and STI testing is less established and pre-existing testing rates are lower. Wider use of sexual health nurses within general practices could result in substantial improvements in HIV and STI screening of MSM in the community.

## Abbreviations

MSM: Men who have sex with men; HIV: Human Immunodeficiency Virus; STIs: Sexually Transmitted Infections; GP: General Practitioner; EIA: Enzyme-linked Immunoassay.

## Competing interests

The authors declared that they have no competing interest.

## Author contributions

AFS oversaw the implementation and management of the nurse; contributed to the design of the study; was primarily responsible for data acquisition, analysis, and interpretation; and for manuscript drafting and preparation. LV made significant contributions to data acquisition, analysis, and interpretation; and help draft the manuscript. CKF conceived the study design and provided manuscript revision. CE provided data and manuscript revision. RC contributed to study design and manuscript revision. LO enabled the collection and procurement of data; and provided manuscript revision. NR enabled the collection and procurement of data and provided manuscript revision. MEH provided data and manuscript revision. MYC contributed to study design, provided supervision to AS; and reviewed the manuscript. All authors read and approved the final manuscript.

## Pre-publication history

The pre-publication history for this paper can be accessed here:

http://www.biomedcentral.com/1471-2334/13/298/prepub
